# Induction of Type I Interferon through a Noncanonical Toll-Like Receptor 7 Pathway during Yersinia pestis Infection

**DOI:** 10.1128/IAI.00570-17

**Published:** 2017-10-18

**Authors:** Miqdad O. Dhariwala, Rachel M. Olson, Deborah M. Anderson

**Affiliations:** Department of Veterinary Pathobiology, University of Missouri College of Veterinary Medicine, Columbia, Missouri, USA; University of California, Davis

**Keywords:** Yersinia pestis, TLR7, type I interferon, YopJ, plague, IFN-β, MyD88

## Abstract

Yersinia pestis causes bubonic, pneumonic, and septicemic plague, diseases that are rapidly lethal to most mammals, including humans. Plague develops as a consequence of bacterial neutralization of the host's innate immune response, which permits uncontrolled growth and causes the systemic hyperactivation of the inflammatory response. We previously found that host type I interferon (IFN) signaling is induced during Y. pestis infection and contributes to neutrophil depletion and disease. In this work, we show that type I IFN expression is derived from the recognition of intracellular Y. pestis by host Toll-like receptor 7 (TLR7). Type I IFN expression proceeded independent of myeloid differentiation factor 88 (MyD88), which is the only known signaling adaptor for TLR7, suggesting that a noncanonical mechanism occurs in Y. pestis-infected macrophages. In the murine plague model, TLR7 was a significant contributor to the expression of serum IFN-β, whereas MyD88 was not. Furthermore, like the type I IFN response, TLR7 contributed to the lethality of septicemic plague and was associated with the suppression of neutrophilic inflammation. In contrast, TLR7 was important for defense against disease in the lungs. Together, these data demonstrate that an atypical TLR7 signaling pathway contributes to type I IFN expression during Y. pestis infection and suggest that the TLR7-driven type I IFN response plays an important role in determining the outcome of plague.

## INTRODUCTION

Type I interferon (IFN) is expressed following the detection of intracellular invasion by host cytosolic or membrane-bound pattern recognition receptors (PRRs) and can lead to the expression of more than 3,000 interferon-stimulated genes (1). The expression of type I IFN can be induced by a number of signaling pathways that become activated upon the cytosolic or vacuolar detection of pathogen-associated molecular patterns (PAMP), including microbial nucleic acids and cell wall or membrane components. Six different Toll-like receptors (TLRs) have been shown to localize to vacuolar membranes following phagocytosis, and when activated, they induce a signal transduction pathway that leads to increases in the expression levels of type I IFN and other cytokines (2). Several of these cytokines, including TLR3, -7, -9, and -13, recognize nucleic acids that can be released when bacteria or viruses are lysed or uncoated in acidified phagolysosomes. Upon bacterial lysis, DNA, single-stranded RNA (ssRNA), and even short double-stranded RNA (dsRNA) are released, which should theoretically activate all of these nucleic acid TLRs, yet generally, *in vivo*, the absence of just one PRR can result in a loss of type I IFN signaling (3). This indicates that there is little redundancy between the TLRs and their roles during infection.

TLR7 and TLR9 are activated by ligand binding, dimerization, and proteolysis in the acidic environment of the phagolysosome (4). These events result in the binding of TLR7 or -9 to its adaptor protein myeloid differentiation factor 88 (MyD88) via their respective TIR (Toll/interleukin receptor) domains. The subsequent formation of a cytosolic protein complex, known as the Myddosome, activates a phosphorylation cascade that leads to the strong activation of inflammatory cytokines and type I IFNs (5). The expression of type I IFN through phagosomal TLR3 and TLR4 requires an alternate adaptor, TIR domain-containing adaptor inducing interferon beta (TRIF), which leads to the high-level expression of IFN-β (6). Nearly all cells in the body express the type I IFN receptor, which induces an antiviral response and the expression of proinflammatory cytokines upon binding to IFN. Type I IFN signaling is frequently detrimental to the host during bacterial infection, with multiple mechanisms being able to cause IFN-dependent immunopathology (7, 8).

We have previously shown that type I IFN signaling contributes to the pathogenesis of plague, a flea-borne disease that has been responsible for three worldwide pandemics (9, 10). Exposure to its causative agent, Yersinia pestis, results in uncontrolled bacterial growth in primary tissues that is accompanied by dissemination through the vasculature and secondary colonization throughout the body (11). Early after infection, Y. pestis establishes an immune-suppressive environment, which requires two virulence factors that are induced in the 37°C host environment. The thermal downregulation of bacterial LpxL causes the hypoacylation of lipid A, resulting in the expression of predominantly the tetra-acylated form that is less stimulatory to mammalian TLR4 (12, 13). In addition, extracellular bacteria express the type III secretion system (T3SS) to inject immune modulators, collectively known as Yersinia outer proteins (Yops), into phagocytic cells (14). Yop activity can inhibit phagocytosis, suppress inflammatory responses, and induce programmed cell death (15–17). The phagocytosis of Y. pestis by neutrophils leads to the killing of bacteria, whereas bacteria generally survive internalization by macrophages (18).

In spite of this and other mechanisms for preventing phagocytosis, Y. pestis is likely taken up by macrophages *in vivo*, where it survives; replicates in a neutral-pH, Rab1B^+^ vacuolar compartment; and eventually causes lysis of the host cell (19–21). Survival in phagosomes is not dependent on the T3SS, and T3SS activity in the phagosome is minimal (22, 23). The role of intracellular Y. pestis in pathogenesis is not well understood but is believed to be important, as viable intracellular bacteria were reported to be detected in macrophages during the first few days of infection *in vivo* (24). In addition, a Y. pestis strain lacking the 2-component regulatory pathway PhoPQ, which is known to severely reduce intracellular survival, was found to be attenuated for virulence in the murine plague model (25–27).

Mice lacking the type I IFN receptor IFNAR were previously shown to be more resistant to pulmonary Y. pestis infection (9). *Ifnar*^−/−^ mice initially developed systemic infection with an inflammatory cytokine profile similar to that of wild-type (WT) mice. In the later stages, mutant mice developed large neutrophilic inflammatory foci in the liver that correlated with the eventual clearance of infection, whereas these foci were less frequent and even absent in WT mice. Furthermore, neutropenia was observed in the bone marrow of infected WT mice, while larger neutrophil populations were found in the bone marrow of *Ifnar*^−/−^ mice, suggesting that type I IFN signaling may contribute to systemic neutrophil depletion. In this work, we explored host-pathogen interactions that are responsible for causing the expression of type I IFN during Y. pestis infection. We identified an atypical TLR7 signaling pathway induced by Y. pestis that is an important contributor to IFN-β expression and characterized its role in the progression of plague.

## RESULTS

### TLR7 is required for optimal expression of IFN-β during Y. pestis infection of macrophages.

**FIG 1:**
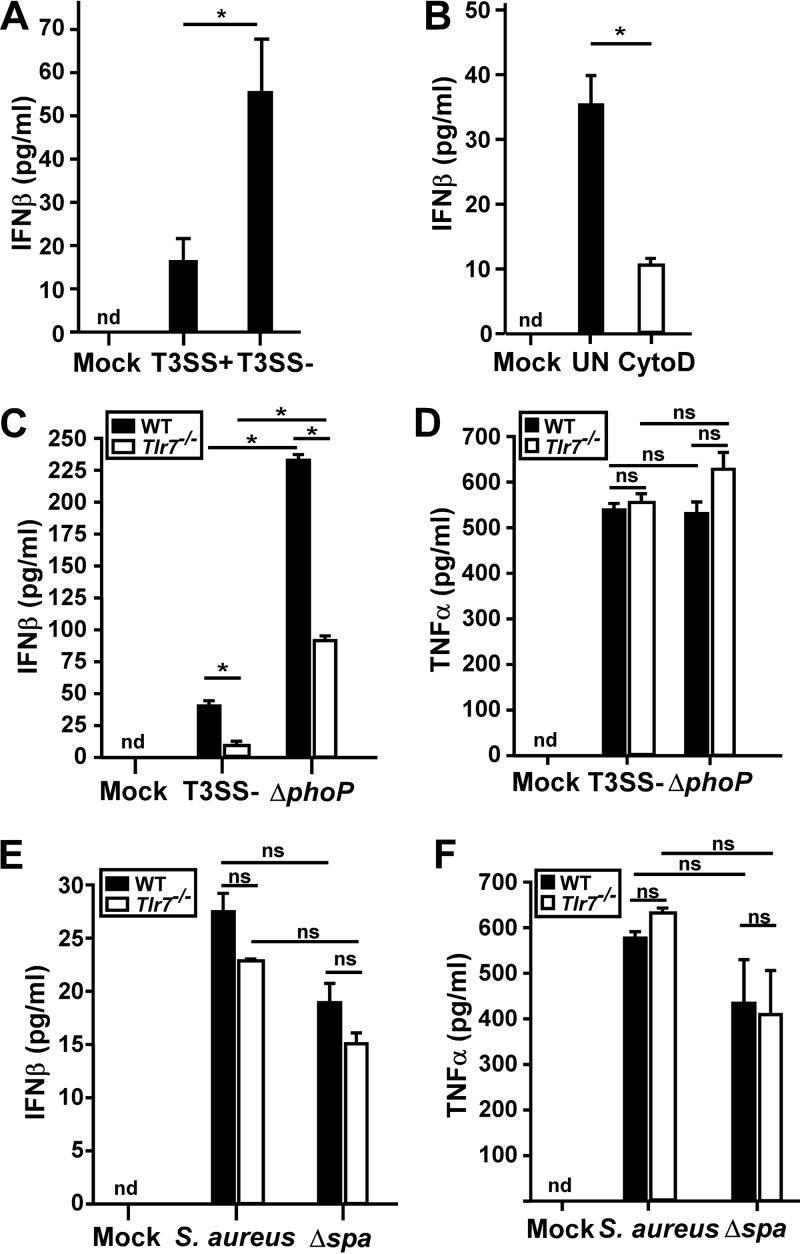
TLR7 is required for optimal IFN-β expression by macrophages infected with Yersinia pestis. (A) WT BMDMs were infected with Y. pestis T3SS^+^ or T3SS^−^ bacteria (both *pgm* negative) at an MOI of 20; the IFN-β level in the supernatant was measured by an ELISA at 4 hpi. (B) RAW 264.7 macrophages were pretreated with cytochalasin D (CytoD) or the vehicle (UN) and then infected with the Y. pestis T3SS^−^ strain, and the IFN-β level in the supernatant was measured at 4 hpi. (C to F) WT or *Tlr7*^−/−^ BMDMs were infected with the Y. pestis T3SS^−^ strain (C and D) or Staphylococcus aureus (E and F) at an MOI of 20 (Y. pestis) or an MOI of 5 (S. aureus), control cells were mock infected, and IFN-β (C and E) and TNF-α (D and F) levels in the supernatant were measured by an ELISA at 4 hpi. Error bars show standard deviations from the means. Data shown are representative of results from at least two independent trials; each sample was tested in duplicate. Data were analyzed by two-way ANOVA followed by the Holm-Sidak test for multiple comparisons. *, *P* < 0.05; ns, not significant; nd, not detected.

To characterize the mechanism by which Y. pestis induces the expression of type I IFN, we measured the secretion of IFN-β induced by T3SS-positive (T3SS^+^) and T3SS-negative (T3SS^−^) bacteria. Bone marrow-derived macrophages (BMDMs) isolated from wild-type C57BL/6 mice produced IFN-β when infected with Y. pestis T3SS^+^ strain KIM D27, and even more IFN-β was induced by T3SS^−^ strain KIM6 (Fig. 1A). IFN-β expression was decreased when macrophages were pretreated with cytochalasin D followed by Y. pestis infection (Fig. 1B; see also Fig. S1A in the supplemental material). This suggests that the inhibition of phagocytosis reduced the expression of type I IFN, indicating that expression occurs following the recognition of intracellular bacteria. Consistent with this hypothesis, L929 cells, a nonphagocytic fibroblast cell line commonly used for measuring IFN-β responses to intracellular bacteria, did not express detectable type I IFN or the interferon-stimulated gene *Ccl5* (Fig. S1B).

Because Y. pestis is believed to survive and replicate in an intracellular phagosomal compartment in macrophages, phagosomal TLRs, rather than cytosolic PRRs, are likely to be activated. We therefore tested for a role for one of these TLRs, TLR7, which recognizes short single-stranded RNA ligands in phagosomes and can be activated by bacterial mRNA (3, 28). Y. pestis-infected BMDMs from *Tlr7*^−/−^ mice expressed very little IFN-β, only about 10% of that of WT BMDMs infected with T3SS^−^ strain KIM6, suggesting that TLR7 is required for the full induction of type I IFN (Fig. 1C). We also tested KIM6Δ*phoP*, which is rapidly lysed following phagocytosis because it fails to prevent the acidification and subsequent maturation of the phagosome. As expected, the Δ*phoP* intracellular survival mutant (T3SS^−^) induced a significant increase in the number of WT BMDMs (Fig. 1C). Similarly, *Tlr7*^−/−^ BMDMs also expressed higher levels of IFN-β when infected with the Δ*phoP* mutant, with an approximately 9-fold increase over that induced by infection with KIM6, suggesting that one or more other PRRs are activated by the Δ*phoP* mutant. Therefore, the importance of TLR7 for the induction of IFN-β expression may be more critical during infection by Y. pestis strains that survive in the intracellular compartment than during infection by bacteria that are killed there. In contrast, *Tlr7*^−/−^ BMDMs secreted similar levels of tumor necrosis factor alpha (TNF-α) during infection with KIM6 or the Δ*phoP* mutant, suggesting that TNF-α secretion by infected macrophages occurs through a different pathway that is neither dependent on TLR7 nor significantly impacted by bacteria that lyse in the intracellular compartment (Fig. 1D).

To verify that *Tlr7*^−/−^ BMDMs were not generally deficient in inducing IFN-β, we infected these cells with Staphylococcus aureus, which is known to activate IFN-β expression through TLR9 (29). Wild-type S. aureus Newman induced IFN-β in WT and *Tlr7*^−/−^ macrophages, with a small, <20%, decrease being observed in the absence of TLR7 (Fig. 1E). When a multiple-comparisons statistical analysis was performed, this difference, although reproducible, was not significant. This result suggests that TLR7 may make a small contribution to IFN-β expression during S. aureus infection of macrophages. The S. aureus Δ*spa* mutant, which lacks protein A, was previously shown to be defective for inducing the expression of IFN-β during dendritic cell infection (30). We therefore also compared IFN-β expression levels between WT and *Tlr7*^−/−^ BMDMs following infection with the S. aureus Δ*spa* mutant. WT and *Tlr7*^−/−^ BMDMs expressed approximately 30% less IFN-β during infection by the Δ*spa* mutant than during infection by WT S. aureus Newman, but this difference was not significant. Importantly, no detectable differences in IFN-β expression levels were detected between WT and *Tlr7*^−/−^ BMDMs infected with the S. aureus Δ*spa* mutant. Likewise, TNF-α expression was decreased only 25% between WT and *Tlr7*^−/−^ BMDMs infected with the S. aureus Δ*spa* mutant, a difference that was not statistically significant (Fig. 1F). This suggests that TLR7 is dispensable for TNF-α and IFN-β secretion during S. aureus infection of macrophages. Overall, the data support a model whereby TLR7 is activated by Y. pestis but not by S. aureus.

### The canonical TLR7 adaptor MyD88 is dispensable for IFN-β expression by Y. pestis-infected macrophages.

**FIG 2:**
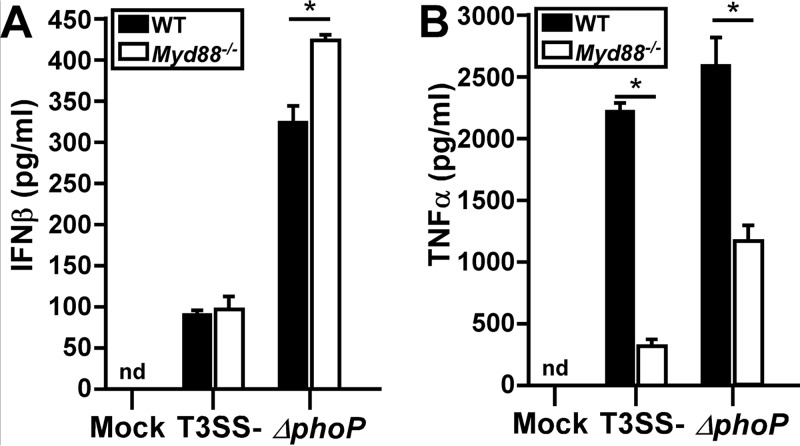
MyD88 is not required for Yersinia pestis induction of IFN-β. Wild-type and *Myd88*^−/−^ BMDMs were infected with the Y. pestis T3SS^−^ strain, and the IFN-β (A) or TNF-α (B) level was measured at 4 hpi. Error bars show standard deviations from the means. Data shown are representative of results from two independent trials; each sample was tested in duplicate. Data were analyzed by two-way ANOVA followed by the Holm-Sidak test for multiple comparisons. *, *P* < 0.05; nd, not detected.

The typical consequence of TLR7 activation is the recruitment of its canonical adaptor MyD88 and the subsequent signal transduction pathway that leads to the activation of the transcription of cytokines, including type I interferon (21). Given that the T3SS is relatively inactive from the intracellular niche, we restricted our *in vitro* studies to using T3SS-deficient strains and asked if MyD88 is involved in IFN-β secretion during Y. pestis infection. BMDMs from C57BL/6 and *Myd88*^−/−^ mice were infected with Y. pestis KIM6, and IFN-β levels were measured by an enzyme-linked immunosorbent assay (ELISA). Unexpectedly, infected *Myd88*^−/−^ BMDMs secreted levels of IFN-β equivalent to those secreted by infected wild-type cells (Fig. 2A). Similarly to KIM6, infection by the Δ*phoP* mutant did not reduce IFN-β expression by *Myd88*^−/−^ BMDMs, indicating that IFN-β secretion by the intracellular survival mutant is also MyD88 independent. In contrast, TNF-α secretion in KIM6-infected macrophages was dependent on MyD88, while *Myd88*^−/−^ BMDMs produced smaller, but still substantial, amounts of TNF-α when infected by the Δ*phoP* mutant (Fig. 2B). These results demonstrate that MyD88 is involved in the expression of proinflammatory cytokines during Y. pestis infection but does not contribute significantly to IFN-β expression. Combined, the data suggest that TLR7 activates IFN-β expression independently of its canonical adaptor MyD88 during Y. pestis infection of macrophages.

### YopJ_KIM_ suppresses IFN-β expression during Y. pestis infection of macrophages.

**FIG 3:**
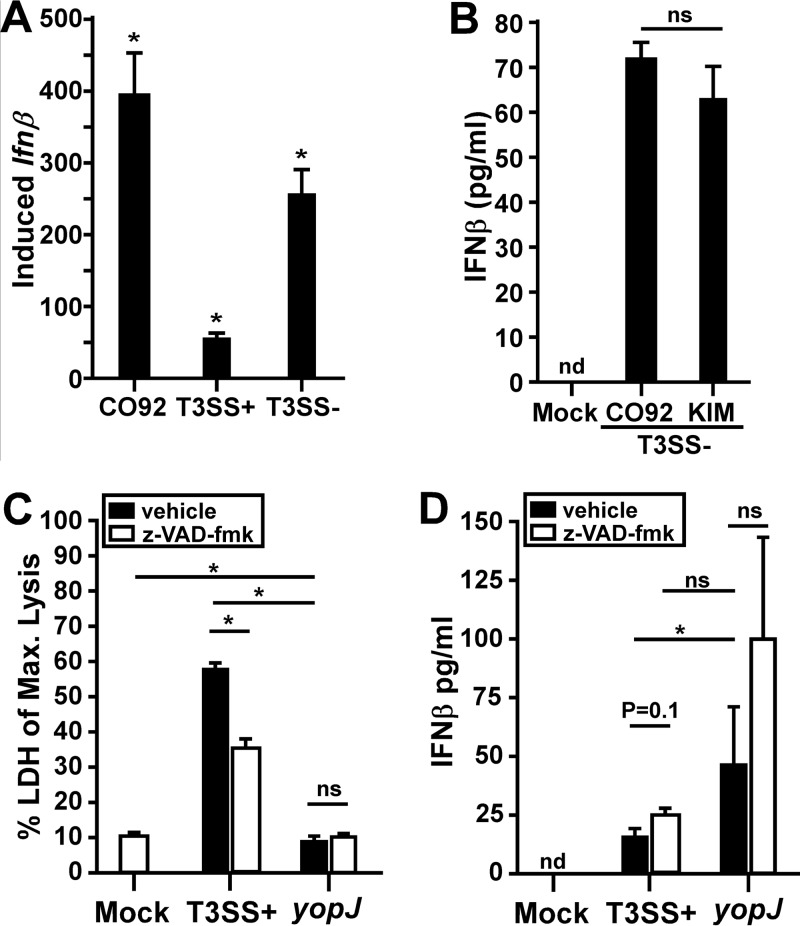
Y. pestis KIM YopJ partially inhibits IFN-β expression. RAW 264.7 macrophages were infected with the virulent wild-type CO92 (T3SS^+^ *pgm*^+^) (A), KIM D27 (T3SS^+^ *pgm*-negative) (A, C, and D), CO92pCD1^−^ (T3SS^−^ *pgm*-negative) (B), or KIM6 (T3SS^−^ *pgm*-negative) (A and B) Y. pestis strain at an MOI of 20. Mock cells were not infected. The *Ifn*β mRNA level was quantified by real-time PCR at 2 hpi (A), or the IFN-β protein level was quantified by an ELISA at 4 hpi (B to D). (C and D) RAW 264.7 macrophages were pretreated with 20 μM z-VAD-fmk or the vehicle control prior to infection with Y. pestis KIM D27 or the *yopJ*_C172A_ mutant at an MOI of 20. At 4 hpi, LDH (C) and IFN-β (D) levels in the supernatant were measured, IFN-β levels were normalized to the amount present in 4 × 10^5^ macrophages (equal to the average number of viable cells in the WT-infected samples measured in panel C), and data shown are representative of results from three independent trials, each with biological replicates that were analyzed in duplicate. Statistical significance was evaluated by one-way ANOVA followed by Sidak's (A) or Tukey's (C and D) test for multiple comparisons or by paired Student's *t* test (B). ns, not significant; *, *P* < 0.05; nd, not detected.

We asked whether wild-type Y. pestis CO92 (T3SS^+^ *pgm*^+^) induced macrophages to transcribe *Ifn*β mRNA. As expected, CO92 induced a large increase in *Ifn*β transcription (Fig. 3A). Unexpectedly, however, the amount of *Ifn*β mRNA induced by CO92, which expresses the T3SS like the KIM D27 strain, was 7-fold larger than that induced by KIM D27 and was even slightly larger than that induced by T3SS^−^ strain KIM6. Similar results were observed when we compared IFN-β secretion by macrophages infected with Y. pestis CO92 (T3SS^+^ *pgm*^+^) and KIM6 (T3SS^−^ *pgm* negative) (see Fig. S2A in the supplemental material). These data suggest that there could be differences in IFN-β expression caused by infection by the two Y. pestis biovars Y. pestis bv. mediaevalis (KIM) and Y. pestis bv. orientalis (CO92). When IFN-β expression levels were compared between macrophages infected with KIM6 and those infected with CO92pCD1^−^ (both T3SS^−^), there was no detectable difference (Fig. 3B). These data strongly suggest that the T3SS of KIM suppresses IFN-β expression, whereas the T3SS of CO92 does not. Consistent with this hypothesis, we found an increase in IFN-β expression when we treated T3SS^+^ KIM-infected macrophages with gentamicin, which killed extracellular (T3SS-active) but not intracellular (T3SS-inactive) bacteria (Fig. S2B) (31). In contrast, gentamicin-treated macrophages infected with the T3SS^−^ strain had no change in IFN-β expression compared to vehicle-treated cells.

One notable sequence difference between the T3SSs of KIM and CO92 is the effector protein YopJ. YopJ from the KIM strain has been shown to have increased activity compared to that of the CO92 strain (32). YopJ is an acetyltransferase that modifies and inactivates host mitogen-activated protein (MAP) kinases, including those that regulate cell death via caspase-1 or those that activate the transcription factors NF-κB, AP-1, and IFN regulatory factor 3 (IRF3) (33–35). We therefore sought to determine if YopJ could suppress the Y. pestis-induced expression of IFN-β. Macrophages were infected with Y. pestis KIM5pCD1Ap^R^ (T3SS^+^ *pgm* negative) expressing a catalytically inactive form of YopJ (YopJ_C172A_) that is injected into the host cell along with all the other Yop effectors (36). Given the impact of YopJ on inducing the rapid lysis of macrophages, we measured lactate dehydrogenase (LDH) release as an indication of host cell lysis and evaluated the effect of inhibition of programmed cell death on IFN-β expression induced by T3SS^+^ Y. pestis. Pretreatment of macrophages with the pancaspase inhibitor z-VAD-fmk (benzyl oxycarbonyl-Val-Ala-Asp OMe-fluoromethylketone) was used to inhibit programmed cell death via caspase-1 or caspase-3 (37). Cells were then infected with Y. pestis or the *yopJ*_C172A_ strain and tested for LDH release as well as IFN-β secretion. As expected, KIM D27 induced rapid lysis of macrophages, and an average of approximately 55% of the maximum possible LDH release occurred at 4 h postinfection (hpi) (Fig. 3C). In contrast, the *yopJ*_C172A_ mutant induced only 10% lysis in this time frame, similarly to cells that were not infected. Treatment of macrophages with z-VAD-fmk caused a reduction in LDH release, indicating that the z-VAD-fmk treatment partially blocked cell death. In contrast, z-VAD-fmk treatment caused no change in LDH release by cells infected with the *yopJ*_C172A_ mutant. We also assayed culture supernatants for IFN-β and normalized the values according to the number of viable cells present. Both WT Y. pestis and the *yopJ*_C172A_ mutant induced IFN-β, with significantly higher levels being found in the *yopJ* mutant-infected samples (Fig. 3D). Together, these data are consistent with a role for YopJ in the suppression of IFN-β expression in addition to its role in inducing the cell death of Y. pestis KIM-infected macrophages. These activities likely combine to cause reduced IFN-β expression by KIM-infected macrophages compared to the CO92 strains.

### TLR7 contributes to pathology in the liver during septicemic plague.

**FIG 4:**
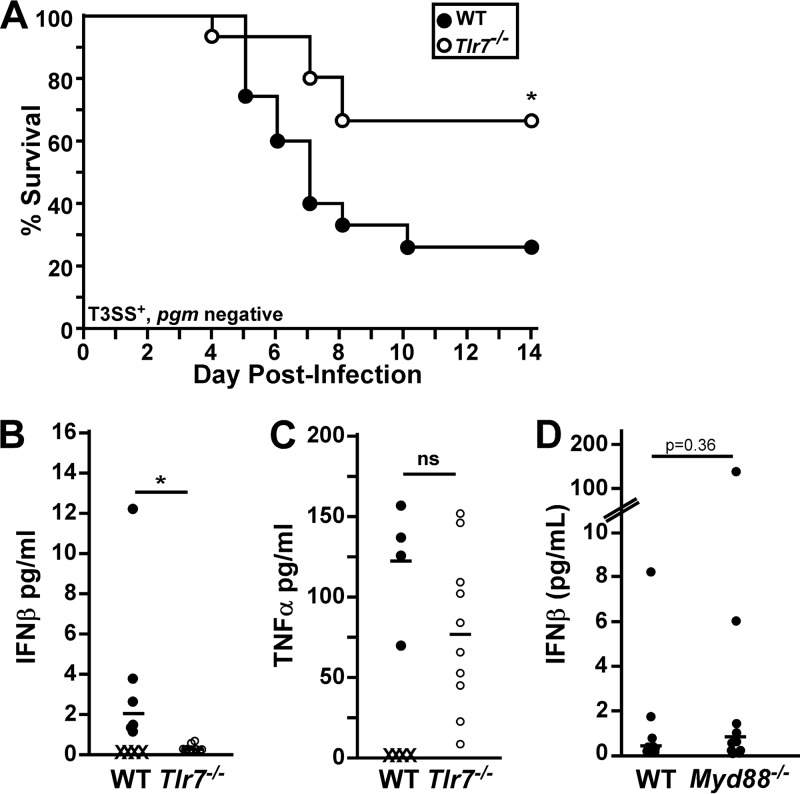
*Tlr7*^−/−^ mice are more resistant to plague and express reduced serum IFN-β levels. Groups of 5 to 8 WT and *Tlr7*^−/−^ mice (A to C) or WT and *MyD88*^−/−^ mice (D) were challenged by intranasal infection with 1 × 10^6^ CFU Y. pestis KIM D27 (T3SS^+^ *pgm* negative) and monitored for survival over 14 days (*n* = 15 per strain, collected in 3 independent trials) (A) or euthanized on day 5 (B and C) or day 3 (D) postinfection, blood was collected, and serum was analyzed for IFN-β (B and D) or TNF-α (C) by an ELISA (*n* = 10 per strain, collected in 2 independent trials). X indicates that the animal died prior to analysis, and bars indicate the medians. Data were analyzed by a Mantel-Cox log rank test (A) or by Student's *t* test (B to D). *, *P* < 0.05.

To determine if TLR7 was important for IFN-β expression *in vivo*, we studied Y. pestis KIM D27 (T3SS^+^ *pgm* negative) infection of *Tlr7*^−/−^ mice. Intranasal challenge of *Tlr7*^−/−^ mice with Y. pestis KIM D27 resulted in increased survival compared to that of WT C57BL/6 mice (Fig. 4A). On day 5 postinfection, serum IFN-β was detectable in WT mice, while *Tlr7*^−/−^ mice had no detectable serum IFN-β (Fig. 4B). In contrast, WT and *Tlr7*^−/−^ mice developed increases in serum TNF-α levels, with no detectable differences between the two groups (Fig. 4C). Also consistent with the *in vitro* data, *Myd88*^−/−^ mice challenged by intranasal infection with Y. pestis KIM D27 developed elevated serum IFN-β levels, with no detectable differences compared to WT mice (Fig. 4D). These data are consistent with a critical role for a noncanonical, MyD88-independent TLR7 pathway in inducing IFN-β expression during Y. pestis infection *in vivo*.

**FIG 5:**
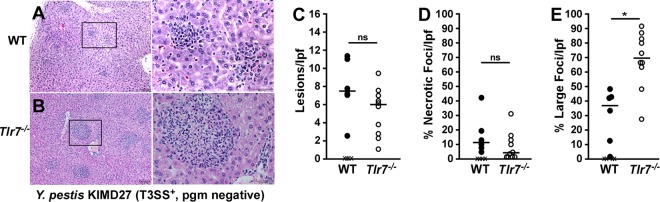
*Tlr7*^−/−^ mice induce granulomatous lesions in the liver during Y. pestis KIM D27 infection. Groups of 5 to 8 WT and *Tlr7*^−/−^ mice were challenged by intranasal infection with Y. pestis KIM D27 (T3SS^+^ *pgm* negative). On day 5 postinfection, animals were euthanized, and formalin-fixed livers were sectioned, stained with hematoxylin and eosin, and analyzed by histopathology. (A and B) Representative lesions for WT (*n* = 6) (A) and *Tlr7*^−*/*−^ (*n* = 10) (B) mice (collected in two independent trials). Boxes outline the zoomed-in areas shown in the bottom panels. Bars, 100 μm (50 μm in the zoomed-in images). (C to E) Quantification of H&E-stained necrotic and inflammatory lesions in the livers from day 5 postinfection showing the number of inflammatory (C) or necrotic (D) lesions per low-power field (lpf) (left) and the percentage of inflammatory foci that were >100 μm in diameter (E). Data were collected in two independent trials and evaluated by unpaired Student's *t* test. *, *P* < 0.05; ns, not significant.

We previously showed that *Ifnar*^−/−^ mice challenged with Y. pestis KIM D27 had larger granulomatous lesions in the livers on day 5 following intranasal infection with the Y. pestis T3SS^+^ *pgm*-negative strain (9). This phenotype was also seen in *Tlr7*^−/−^ mice. When analyzed by histopathology, the livers of *Tlr7*^−/−^ mice frequently developed very large (100- to 200-μm diameter), granulomatous inflammatory foci that were not observed in wild-type mice (Fig. 5A to E). These data are consistent with an improved neutrophilic response in *Tlr7*^−/−^ mice. As expected for the Y. pestis *pgm*-negative strain, the lungs of WT and *Tlr7*^−/−^ mice generally had mild pathology, with the occasional appearance of inflammatory foci (see Fig. S3A and S3B in the supplemental material). In contrast, the spleens of WT mice generally contained large amounts of red pulp necrosis and bacteria, whereas this was less severe in *Tlr7*^−/−^ mice (Fig. S3C and S3D). Combining the histopathology and cytokine data, the phenotype of *Tlr7*^−/−^ mice during Y. pestis KIM D27 infection suggests a lack of type I IFN signaling (9).

**FIG 6:**
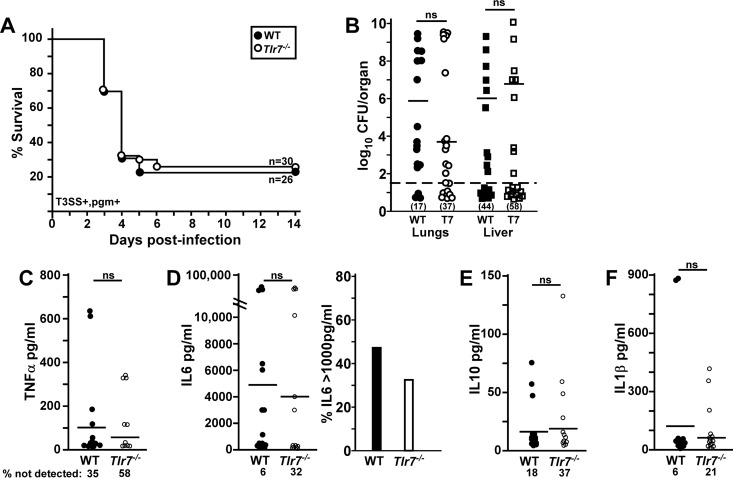
*Tlr7*^−/−^ mice experience reduced serum TNF-α levels and sepsis during Y. pestis CO92 infection. Groups of 10 WT and *Tlr7*^−/−^ mice were challenged by intranasal infection with 1 × 10^3^ CFU Y. pestis CO92 (T3SS^+^ *pgm*^+^). (A) Survival was monitored over 14 days. Data shown were collected in 3 independent trials (*n* = 26 or 30 per group). (B to F) On day 3 postinfection, animals were euthanized; blood was collected by cardiac puncture for multiplex serum cytokine analysis; and lungs, liver, and spleen were homogenized for the enumeration of the bacterial load. (B) Bacterial counts in the indicated tissues. Bars indicate medians, and the percentages of each tissue that had undetectable bacterial titers are indicated in parentheses. (C) Serum TNF-α. (D) IL-6. The right panel shows percentages of animals in each group with high IL-6 titers. (E) IL-10. (F) IL-1β. Bars indicate the means, and the percentages of mice with undetectable cytokines are indicated (*n* = 17 WT and 19 *Tlr7*^−/−^ mice [3 WT mice and 1 *Tlr7*^−/−^ mouse died prior to 72 hpi], collected in two independent trials). Statistical significance was evaluated by a Mantel-Cox log rank test (A) or by Student's *t* test (B to F). ns, not significant.

To gain insight into the function of this pathway during lung infection, we also characterized the phenotype of *Tlr7*^−/−^ mice during respiratory infection with Y. pestis CO92, a *pgm*^*+*^ strain that causes fulminant bronchopneumonia along with secondary sepsis (11). WT and *Tlr7*^−/−^ mice were challenged by intranasal infection with 1 × 10^3^ CFU (approximately 2.5× the 50% lethal dose [LD_50_]) of Y. pestis CO92. A small, but not significant, increase in the survival of *Tlr7*^−/−^ mice was observed (Fig. 6A). Moribund animals and those found dead in the survival studies were examined postmortem for lesions and bacterial growth in the lungs and liver in order to determine the extent of primary pneumonic plague and secondary septicemic plague. All of the animals in both groups developed severe red pulp necrosis in the spleen along with widespread bacteria, indicating systemic infection in 100% of the mice (data not shown). Approximately 38% of WT mice developed primary bronchopneumonia, while 62% had interstitial pneumonia (Table 1). In contrast, 69% of *Tlr7*^−/−^ mice had moderate to severe focal bronchopneumonia, with large areas of neutrophilic infiltrates and bacterial growth in the alveolar space, while other sections of the lung were unaffected. This suggests that *Tlr7*^−/−^ mice were more susceptible to primary pneumonia. In the liver, WT mice had significantly increased pathology and apparent increases in the size and frequency of bacterial colonies compared to *Tlr7*^−/−^ mice (Table 1). This suggests that disease in the liver was less severe and perhaps that there was a more effective clearance of bacteria.

**TABLE 1:** Pathology summary for moribund mice^*e*^

Group^*a*^	% bronchopneumonia^*b*^	Mean liver severity score ± SD^*c*^	Mean liver bacterium score ± SD^*d*^
WT	38	4.3 ± 0.7	3.3 ± 0.6
*Tlr7*^−/−^	69	3.1 ± 0.9***	2.6 ± 0.7*

a *n* = 13 WT mice and 16 *Tlr7*^−/−^ mice, collected in two independent trials.

b Defined as bacterial colonies in alveoli, neutrophilic inflammation, and congestion with or without hemorrhage.

c Includes necrosis and inflammatory infiltrates (8 was the highest possible score).

d Bacterial colonies in the liver, visible by H&E stain, were scored for the frequency and size of the colonies (4 was the highest possible score).

e ***, *P* < 0.001; *, *P* < 0.05 (analyzed by an unpaired *t* test).

We also examined bacterial growth and the inflammatory response at 72 hpi in the lungs, liver, and spleen but found no detectable differences in the amounts of bacteria that could be recovered from any of the organs (Fig. 6B and spleen data not shown). However, we could not recover bacteria from a higher percentage of *Tlr7*^−/−^ mice than of WT mice. Serum TNF-α, interleukin-6 (IL-6), IL-1β, IL-10, and IFN-γ levels were also not detectably different (Fig. 6C to F and IFN-γ data not shown). Again, a higher percentage of *Tlr7*^−/−^ mice consistently harbored undetectable TNF-α, IL-6, IL-10, and IFN-γ, and fewer *Tlr7*^−/−^ mice had high IL-6 titers (>1,000 pg/ml) than WT mice (Fig. 6D). From this analysis, it appears there could be early bacterial clearance in the lungs of *Tlr7*^−/−^ mice that may directly or indirectly impact bacterial dissemination and the onset of sepsis. This is somewhat contradictory to the histopathology data shown in Table 1, which show that *Tlr7*^−/−^ mice are more susceptible to pneumonia.

**FIG 7:**
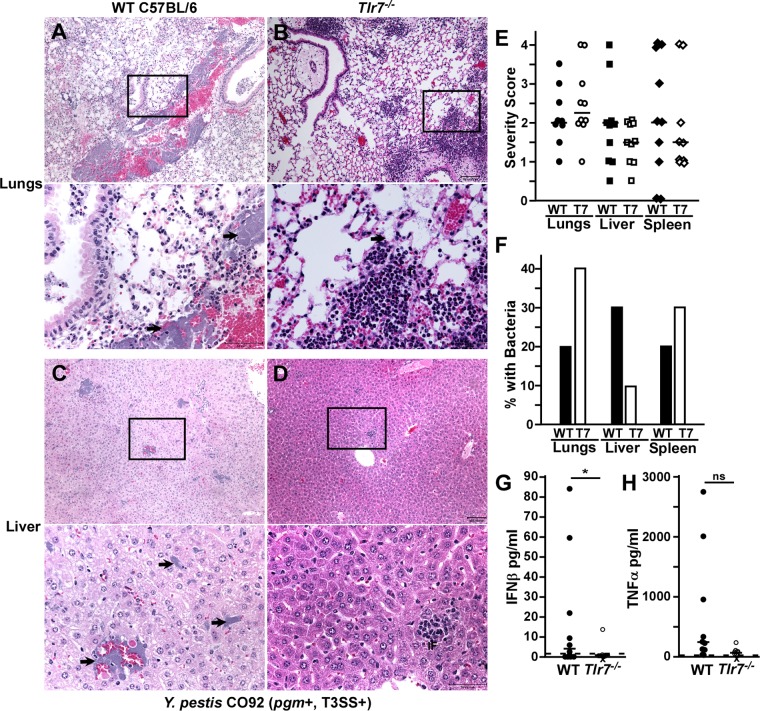
TLR7 is associated with increased liver pathology during pneumonic plague. (A to F) Groups of 5 WT (A, C, E, and F) or *Tlr7*^−/−^ (B, D, E, and F) mice were challenged by intranasal infection with 1 × 10^3^ CFU Y. pestis CO92 (T3SS^+^ *pgm*^+^). (A to D) On day 3 postinfection, animals were euthanized, and formalin-fixed lungs (A and B), liver (C and D), and spleen (see Fig. S4A and S4B in the supplemental material) were sectioned, stained with hematoxylin and eosin, and analyzed by histopathology. Representative lesions are shown; boxes outline the zoomed-in areas shown in the bottom panels, and arrows point to bacteria in the zoomed-in images. Bars, 100 μm (50 μm in the zoomed-in images). (E and F) Quantification of histopathology. (E) Severity scoring; (F) percentages of samples with bacterial microcolonies visible by histopathology. Bars indicate medians. (G and H) Serum IFN-β and TNF-α levels were measured by an ELISA. Bars indicate medians, dotted lines indicate the limit of detection, and X indicates that the animal died prior to analysis. Statistical significance was evaluated by Student's *t* test. *, *P* < 0.05. Data shown in all panels were collected in 2 independent trials (*n* = 10 per group).

We therefore also examined pathology at 72 hpi. At this time point, 20% of WT mice and 40% of *Tlr7*^−/−^ mice had developed bronchopneumonia, characterized by alveolar necrosis, neutrophilic infiltrates, hemorrhage, exudates, edema, and bacterial colonies (Fig. 7A and B show representative lesions) (11, 38). The *Tlr7*^−/−^ group had a higher median severity score, with increased neutrophilic inflammation in the lungs (Fig. 7E). This is consistent with the above-described data suggesting that *Tlr7*^−/−^ mice are more susceptible to bronchopneumonia. In the liver, WT mice harbored frequent bacterial colonies, with moderate lesions of hemorrhage and inflammation (Fig. 7C, E, and F). In contrast, livers from *Tlr7*^−/−^ mice had a lower median severity score, with frequent small inflammatory foci as well as occasional bacteria and mild hemorrhage (Fig. 7D to F). Pathologies in the spleen were not detectably different between WT and *Tlr7*^−/−^ mice (Fig. 7E and F and Fig. S4). These data are consistent with a liver-specific role for TLR7 that promotes disease in this tissue. In addition, serum IFN-β was nearly absent in *Tlr7*^−/−^ mice, with a significant reduction compared to WT mice, while serum TNF-α levels were not detectably different (Fig. 7G and H). Overall, the phenotypic observations suggest a bifunctional, tissue-specific role for TLR7 in the host response to Y. pestis CO92 infection.

## DISCUSSION

Yersinia pestis evades the activation of TLR-mediated inflammatory responses due primarily to three mechanisms: the lack of flagellin expression, downregulation of hexa-acylated lipid A, and injection of effector Yops by the T3SS (12, 39). Here we showed that in spite of these evasive mechanisms, TLR7 recognition of Y. pestis occurs and leads to the expression of type I IFN. We observed a TLR7-dependent elevation of serum IFN-β levels in murine models of pneumonic and septicemic plague, and *Tlr7*^−/−^ mice appeared to have controlled disease in the liver, probably through a more effective neutrophil response. Combined with the previously reported phenotype of *Ifnar*^−/−^ mice, there is strong evidence that TLR7-driven type I IFN induces immunopathology during plague.

Type I IFN is known to have differential effects on innate and adaptive immune responses that can be influenced by disease progression, making the role of this cytokine subject to context- and tissue-specific changes (40). In mice with TLR7, perhaps an early type I IFN response that dampened neutrophil recruitment in the lungs reduced tissue damage, which resulted in fewer WT mice developing bronchopneumonia. In *Tlr7*^−/−^ mice, increased numbers of neutrophils may have initially led to improved bacterial clearance, but perhaps the congestion and tissue damage caused by neutrophils ultimately promoted the development of pneumonia. In contrast, the dysregulation of neutrophil recruitment in the liver of WT mice may result in accelerated tissue damage, bacterial growth, and death from septicemic plague. As a consequence of the bifunctional role of TLR7, there was no significant survival difference between WT and mutant mice in the pneumonic plague model.

*In vitro*, WT Y. pestis CO92 (T3SS^+^ *pgm*^+^) induced levels of IFN-β expression from infected macrophages similar to those induced by the attenuated strains lacking the pigmentation locus and/or the type III secretion system. This suggests that the virulence factors used by extracellular wild-type Y. pestis, such as the T3SS, do not prevent IFN-β expression. This can be explained by the activation of TLR7 by intracellular bacteria, perhaps through the release of RNA or another ligand by viable Yersinia bacteria. The Y. pestis *phoP* mutant, which is defective in preventing the acidification of the vacuole and survives poorly in the intracellular compartment, induced a TLR7-independent increase in IFN-β expression. This observation suggests that the *phoP* mutant presents new ligands and/or that the acidification of the phagosome allows for the activation of other TLRs. In either case, it appears that TLR7 is stimulated by WT Y. pestis when other PRRs are not activated.

**FIG 8:**
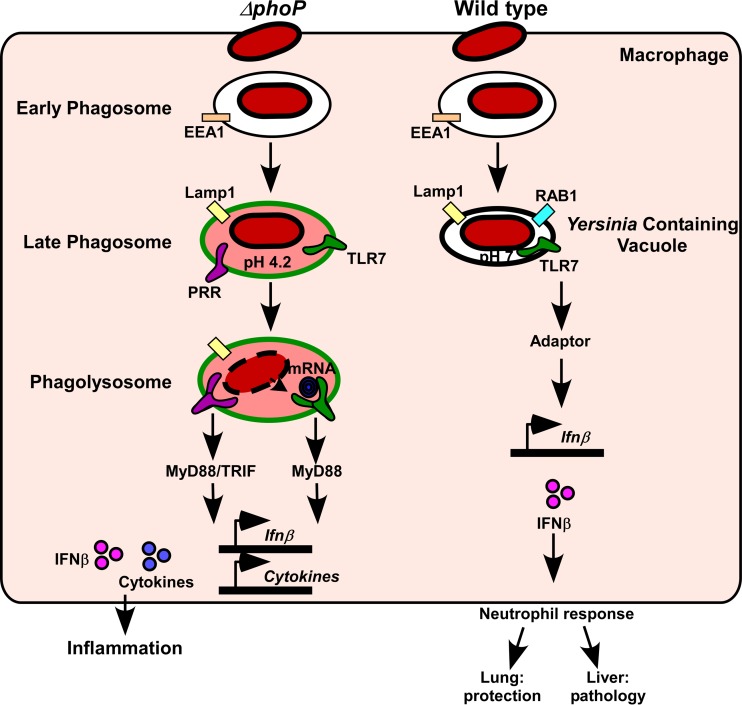
Model for atypical MyD88-independent TLR7 signaling during Yersinia pestis infection. Wild-type Y. pestis prevents the acidification of the macrophage phagosome, and the bacteria remain viable in a pathogen-containing vacuole. For this compartment, we hypothesize that Y. pestis stimulates atypical TLR7 signaling (MyD88 independent), which activates the expression of IFN-β. In contrast, the *phoP* mutant is unable to prevent the acidification and maturation of the phagosome. Consequently, bacteria lyse and likely stimulate canonical TLR signaling through MyD88 and TRIF, which activates the transcription of IFN-β, TNF-α, and other proinflammatory cytokines. During pneumonic plague, the TLR7 type I IFN response protects the lungs but causes pathology in the liver.

Moreover, we found that MyD88 was not required for IFN-β expression, indicating that Y. pestis activation of macrophage TLR7 does not result in its binding to MyD88. Together, these observations suggest a hypothetical model whereby the atypical activation of TLR7 by intracellular Y. pestis allows for the transmission of a MyD88-independent signal that induces IFN-β expression (Fig. 8). In this model, TLR7 might be alternatively activated in the Yersinia-containing vacuole, which has a neutral pH, wherein TLR7 presumably does not undergo dimerization and autoproteolysis. However, it is also plausible that TLR7, as either a homodimer or a heterodimer with another TLR, can signal through a MyD88-independent mechanism by binding to a novel PAMP. Future experiments will address these models in order to understand this atypical pathway and how it functions during bacterial infection.

TLR7 appears to be associated with a suppressed neutrophilic response, yet two outcomes of this were observed. In the lungs, the TLR7 host response played a significant role in preventing the development of bronchopneumonia, while in the liver, the suppression of the neutrophilic response through TLR7 contributed to disease. Whether TLR7 is accidently triggered by the phagocytosis of Y. pestis or is a virulence mechanism, selected for by the unique transmission cycle of plague that permits its worldwide persistence, can be understood through the study of the molecular components of this atypical signaling pathway and its function in innate immunity.

## MATERIALS AND METHODS

### Bacterial strains.

Bacterial strains are listed in Table 2. Yersinia pestis strains were routinely grown fresh from a frozen stock by streaking for isolation onto heart infusion agar (HIA) plates or HIA plates supplemented with 0.005% (wt/vol) Congo red and 0.2% (wt/vol) galactose to screen for bacteria that retain the pigmentation locus (41). Staphylococcus aureus was streaked for isolation on Luria-Bertani (LB) agar. *In vitro* experiments were performed in the nonpigmented (*pgm*-negative) strain background as a model for the wild-type strain. For intranasal challenge studies, a single colony was used to inoculate calcium-supplemented heart infusion broth (Ca-HIB) and grown for 18 to 24 h at 37°C at 125 rpm. All work with wild-type *pgm*^+^ Y. pestis strain CO92 was performed in a select-agent-authorized biosafety level 3 (BSL3) laboratory.

**TABLE 2:** Bacterial strains used in this study

Strain	Phenotype^*a*^	Reference
Yersinia pestis		
Y. pestis bv. orientalis		
CO92	*pgm*^+^ T3SS^+^	48
CO92pCD1^−^	*pgm* negative T3SS^−^	This study
Y. pestis bv. mediaevalis		
KIM D27	*pgm* negative T3SS^+^	49
KIM6^+^	*pgm*^+^ T3SS^−^	50
KIM6^−^	*pgm* negative T3SS^−^	This study
KIM6 *phoP*	*pgm*^+^ T3SS^−^ lacking PhoP	51
KIM6^−^ *yopJ*	*pgm* negative pCD1Ap *yopJ*_C172A_	36
Staphylococcus aureus		
Newman	Wild type (clinical isolate)	52
Δ*spa*	Newman lacking protein A	53

a *pgm*, pigmentation locus (102-kb insertion element in the chromosome); T3SS, type III secretion system (70-kb virulence plasmid pCD1).

### Isolation of the Y. pestis nonpigmented mutant (KIM6) and CO92pCD1.

The spontaneous loss of the pigmentation locus from the Y. pestis KIM6^+^pCD1^−^ strain was identified following plating onto Congo red agar (41, 42). The deletion of the pigmentation locus on the chromosome was verified by restreaking onto Congo red and PCR, as previously described, prior to use in experiments (43). CO92pCD1^−^ was generated from Y. pestis CO92 by using the suicide vector pCVD442 (44). The loss of pCD1 was verified by PCR and phenotypic analysis (data not shown); the strain isolate was approved as exempt from Select Agent and Toxins regulations by the U.S. Centers for Disease Control and Prevention Division of Select Agents and Toxins prior to use in experiments.

### Vertebrate animals: ethics statement.

All animal procedures were performed in compliance with guidelines of the Office of Laboratory Animal Welfare and the National Institutes of Health *Guide for the Care and Use of Laboratory Animals* (45) and were approved by the University of Missouri Animal Care and Use Committee.

C57BL/6J mice were the inbred strain background of *Tlr7*^−/−^ and *Myd88*^−/−^ mice (Jackson Laboratories, Bar Harbor, ME). Wild-type C57BL/6J and mutant strains were bred in-house at the University of Missouri. Male and female wild-type and mutant mice ranging from 15 to 30 g were used for challenge experiments. All infected mice were monitored by daily assignment of health scores, which involved assessments of their appearance and activity. Animals that survived to the end of the 14-day observation period or were identified as moribund (defined by pronounced neurologic signs, inactivity, and severe weakness) were euthanized by CO_2_ asphyxiation followed by bilateral pneumothorax or cervical dislocation, according to the American Veterinary Medical Association Guidelines on Euthanasia (54).

### Histopathology.

Lungs were perfused and fixed in 10% formalin, along with liver and spleen. Organs were further processed for paraffin embedment, blocked in wax, and cut into 5-μm sections. Tissue sections were stained with hematoxylin and eosin (H&E), and coverslips were permanently affixed to stained slides. Sample identities were blinded for analysis. For quantification of the inflammatory lesions in liver, 10 nonoverlapping low-power fields were counted for each animal; two independent counts were taken, and the average of these counts was reported. Severity scoring was based on the sizes of necrotic and inflammatory lesions and their frequencies in the tissue section; each category was scored from 0 to 4, and the sum is reported as a severity score in Table 1.

### Infection studies.

At 72 hpi, mice were euthanized, and blood, lungs, liver, and spleen were collected. Serum was collected following centrifugation, treated with antibiotics to inactivate Y. pestis, and stored at −80°C until samples from both trials could be analyzed together. Serum cytokine levels were measured by a multiplex assay (5 cytokines; Sigma-Millipore) (Fig. 6) or by an ELISA (Fig. 7). Tissues were homogenized in sterile phosphate-buffered saline (PBS) and then diluted and plated onto HIA in duplicate for the enumeration of bacteria.

### Bone marrow-derived macrophage isolation.

Bone marrow was isolated from C57BL/6, *Tlr7*^−/−^, and *Myd88*^−/−^ mice, as previously described, by culturing for 7 days in Dulbecco's modified Eagle's medium (DMEM) containing 20 ng/ml macrophage colony-stimulating factor (M-CSF) and 10% fetal bovine serum (FBS) (eBioscience, San Diego, CA) (46).

### Cellular infection assay.

RAW 264.7 macrophages, BMDMs, or L929 cells were plated at 1 × 10^6^ cells per well in a 12-well plate. Where indicated, cytochalasin D or the pancaspase inhibitor z-VAD-fmk (InvivoGen, San Diego, CA) was added prior to infection. As a control, 4 h of treatment with 1 μg/ml lipopolysaccharide (LPS) from Escherichia coli (Sigma-Aldrich, MO) was used to stimulate IFN-β expression. Unless otherwise indicated, all Yersinia strains were grown at 26°C for 16 to 24 h, diluted 1:10 in fresh medium, and grown at 37°C for 2 to 3 h prior to *in vitro* infection. Staphylococcus strains were grown at 37°C for 16 to 24 h, diluted 1:10 in fresh medium, and grown at 37°C for 2 to 3 h prior to infection. For each experiment, the infection dose was verified by plating in triplicate. To initiate contact, infection mixtures were centrifuged at low speed (41 × *g*) for 5 min at room temperature. At the indicated times postinfection, cells were lysed for RNA extraction, and cell culture supernatants were harvested and stored at −80°C until use, or the IFN-β level in the supernatant was measured by an ELISA (PBL Interferon Source, Piscataway, NJ). Duplicate wells were infected for all trials.

### RNA isolation and real-time PCR.

RNA isolation was performed by using an RNeasy minikit according to the manufacturer's instructions (Qiagen, CA). Total RNA was treated with Turbo DNase (Ambion, TX) to remove residual genomic DNA contamination. First-strand cDNA synthesis was carried out by using Moloney murine leukemia virus reverse transcriptase (MMLV-RT) (Promega, WI) on 2 μg of total RNA according to the manufacturer's instructions. SYBR green PCR master mix (Applied Biosystems, CA) was used along with previously described gene-specific primers for *Ifn*α, *Ifn*β, or *Ccl5* to detect the presence of an amplified product (9). All samples were run in duplicate. Results were analyzed by using relative quantification with 7300 SDS software (Applied Biosystems, CA). Data were normalized to the values for the mouse gene *Ywhaz*, which is constitutively expressed with minimal change (9, 47).

### LDH assay.

RAW 264.7 macrophages were plated at 1 × 10^6^ cells per well in a 12-well plate. Where indicated, the pancaspase inhibitor z-VAD-fmk (InvivoGen, San Diego, CA) was added prior to infection. Cells were infected with the indicated Y. pestis strains at a multiplicity of infection (MOI) of 20, and the dose was verified by plating in triplicate. At 4 h postinfection, supernatants were harvested and analyzed immediately for LDH levels according to the manufacturer's recommendations (Promega, Madison, WI). Mock-infected cells were either lysed by treatment with Triton X-100 to determine maximum LDH release or left untreated for use as a negative control. All samples were run in duplicate. Data are represented as a percentage of maximum lysis.

### Statistical evaluation.

Data from all trials were combined and analyzed for statistical significance. Comparisons of two samples were made by paired Student's *t* test; multiple comparisons were evaluated by one-way analysis of variance (ANOVA) followed by Tukey's or Sidak's posttest. Statistical significance for survival was evaluated by using the Mantel-Cox (log rank) test. Significance was concluded when the *P* value was <0.05.

## Supplementary Material

Supplemental material
