# Supplementary material for: Induction of Type I Interferon through a Noncanonical Toll-Like Receptor 7 Pathway during Yersinia pestis Infection
Source: Infect Immun. 2017 Oct 18;85(11):e00570-17. doi: 10.1128/IAI.00570-17 (PMC5649010; doi:10.1128/IAI.00570-17)
Supplement: Supplemental material [file supp_85_11_e00570-17__index.html]

Supplemental material 

# Induction of Type I Interferon through a Noncanonical Toll-Like Receptor 7 Pathway during Yersinia pestis Infection

## Supplemental material

- Supplemental file 1 -

  Fig. S1. Intracellular *Y. pestis* stimulates IFN-β expression in macrophages. Fig. S2. Extracellular *Y. pestis* KIM suppresses IFN-β expression by macrophages. Fig. S3. Less severe pathology in the spleens of Tlr7−/− mice on day 5 following infection by *Y. pestis* KIM D27. Fig. S4. Similar pathology in the spleens of Tlr7−/− mice on day 3 following infection by *Y. pestis* CO92.

  PDF, 1.4M
