# Supplementary material for: Induction of Type I Interferon through a Noncanonical Toll-Like Receptor 7 Pathway during Yersinia pestis Infection
Source: Infect Immun. 2017 Oct 18;85(11):e00570-17. doi: 10.1128/IAI.00570-17 (PMC5649010; doi:10.1128/IAI.00570-17)

Supplemental Information.

Induction of type I interferon through a non-canonical Toll-like receptor 7 pathway during Yersinia pestis infection

Dhariwala, et al

Supplemental Figure Legends.

Supplemental Figure S1. *Intracellular Y. pestis stimulates IFNβ expression in macrophages.* (A) RAW 264.7 cells were infected with *Y. pestis* T3SS+ or T3SS- followed by treatment with cytochalasin D to block phagocytosis; IFNβ was detected by ELISA at 4 HPI. (B) L929 fibroblasts were infected with *Y. pestis* T3SS+pgm- at MOI of 20 and the indicated mRNA was quantified by real time PCR at 2 HPI. Data shown are representative of 2-3 independent trials; error bars show standard deviation from the mean; data were analyzed by Student's t-test, *P<0.05, nd: not detected.

Supplemental Figure S2. *Extracellular Y. pestis KIM suppress IFNβ expression by macrophages.* RAW 264.7 macrophages were infected with (A) virulent wild type CO92 (T3SS+, pgm+), (B), KIM D27 (T3SS+, pgm-) or (A-B) KIM6 (T3SS-, pgm-) *Y. pestis* at a multiplicity of infection (MOI) of 20; mock cells were not infected. After 30 min, gentamicin was added to kill extracellular bacteria and the intracellular infection continued; IFNβ was quantified in the culture supernatant by ELISA at 4 HPI. Data shown are representative of 2-3 independent trials; error bars show standard deviation from the mean; data were analyzed by Student's t-test, *P<0.05, nd: not detected.

Supplemental Figure S3. *Less severe pathology in the spleens of $Tlr7^{-/-}$ mice on day 5 following infection by Y. pestis KIM D27*. Groups of 5-8 WT (A,C) and $Tlr7^{-/-}$ (B,D) mice were challenged

by intranasal infection with *Y. pestis* KIM D27 (T3SS+pgm-). On day 5 post-infection, animals were euthanized and formalin-fixed lungs (A-B) and spleens (C-D) were sectioned, stained with hematoxylin and eosin and analyzed by histopathology. Representative lesions are shown for WT (n=6) and *Tlr7*$^{-/-}$ (n=10); collected in two independent trials); boxes outline the zoomed in areas shown in the lower panels; arrows point to bacterial microcolonies;N: necrosis, N/I: neutrophilic inflammation, H: hemorrhage, E: exudate. Scale bar indicates 100 µm (50µm in the zoomed-in images).

Supplemental Figure S4. *Similar pathology in the spleens of Tlr7*$^{-/-}$ *mice on day 3 following infection by Y. pestis CO92.* Groups of 5 WT (A) or *Tlr7*$^{-/-}$ (B) mice were challenged by intranasal infection with $1x10^3$ CFU *Y. pestis* CO92 (T3SS+, pgm+). On day 3 post-infection, animals were euthanized and formalin-fixed spleens were sectioned, stained with hematoxylin and eosin and analyzed by histopathology. Representative lesions are shown; boxes outline the zoomed in areas shown in the lower panels. Scale bar indicates 100 µm (50 µm in the zoomed-in images).

Supplemental Fig S1

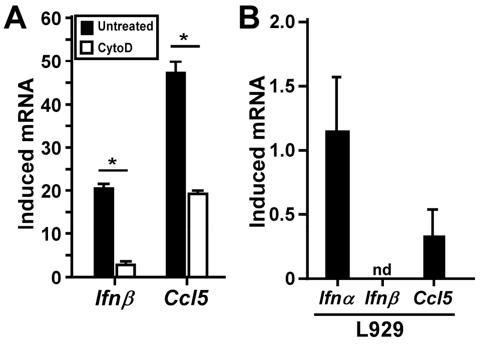

Supplemental Figure S2

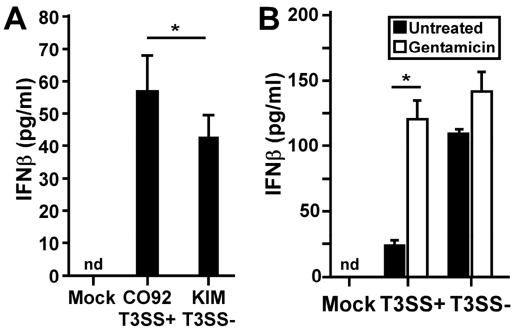

Supplemental Fig S3

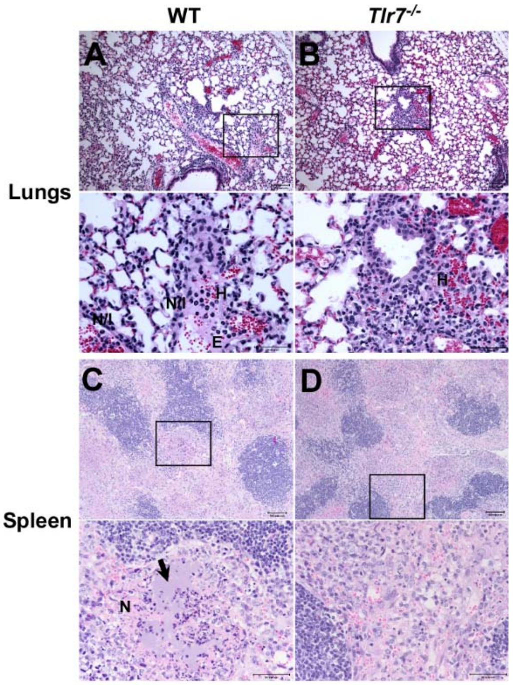

Y. pestis KIM D27 (*pgm*-, T3SS+)

Supplemental Fig S4

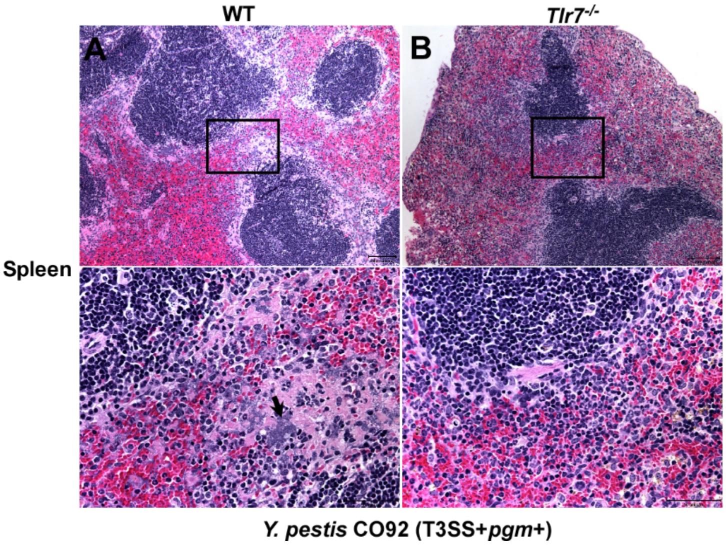

Supplement: Supplemental material [file IAI.00570-17_zii999092200s1.pdf]
